# Stress ulcer prophylaxis in non-critically ill patients: A cross-sectional survey in Saudi Arabia

**DOI:** 10.1097/MD.0000000000046252

**Published:** 2025-11-28

**Authors:** Osamah M. Alfayez, Mashael AlFaifi, Yara Ajeebi, Abdullah A. Alalwan, Eiman Ibrahim, Omar S. Alkhezi

**Affiliations:** a Department of Pharmacy Practice, College of Pharmacy, Qassim University, Qassim, Saudi Arabia; b Pharmaceutical Care Services, King Saud Medical City, Riyadh, Saudi Arabia; c College of Medicine, Jazan University, Jazan, Saudi Arabia; d Department of Clinical Pharmacy, College of Pharmacy, Prince Sattam Bin Abdulaziz University, Al-Kharj, Saudi Arabia.

**Keywords:** cross-sectional study, non-critically ill, Omeprazole, proton pump inhibitors, stress ulcer prophylaxis, survey

## Abstract

Stress ulcer prophylaxis (SUP) use is quite prevalent in the Saudi healthcare system in the general wards as reported by several studies. We aimed to assess the knowledge of physicians and their attitudes toward SUP prescription in non-critically ill patients in Saudi Arabia and identify factors associated with inappropriate SUP use. An electronic cross-sectional survey was administered to physicians registered with the Saudi Commission for Health Specialties. The questionnaire consisted of queries related to demographic information, SUP prescription patterns and frequency, preferred medications, and attitudes toward SUP. A total of 210 respondents completed the survey. Approximately half (48.1%) of the respondents reported prescribing SUP to non-critically ill patients, and 42.8% of respondents thought that their SUP use was supported by evidence. A fear of gastrointestinal (GI) bleeding and safety perceptions were major factors in SUP use. Clinical experience, medical rank, belief in evidence-based use of SUP, and fear of GI bleeding were all significantly correlated with SUP use (*P* <.05). Notably, 79% of respondents reported that they did not receive a pharmacist recommendation to stop therapy, and 38% said they would continue SUP upon discharge. This study presented the perspective of physicians regarding the main reasons for their use of SUP, along with factors significantly associated with this practice. Our findings indicate that there are opportunities to improve physician knowledge and empower interprofessional support to reduce inappropriate SUP use.

## 1. Introduction

A stress ulcer implies a stress-related mucosal injury that may result in bleeding.^[1,2]^ It is known to be associated with negative outcomes in critically ill patients, such as an increase in the length of hospitalization and mortality. Moreover, several risk factors have been reported to increase the risk of stress ulcer-related bleeding. These include recent surgery, mechanical ventilation for more than 48 hours, use of nonsteroidal anti-inflammatory drugs, high-dose corticosteroids, and anticoagulation therapy.^[1,3]^

The use of acid suppressants as stress ulcer prophylaxis (SUP) has been practiced for several years.^[1,3]^ Medications such as proton pump inhibitors (PPIs) are widely utilized in addition to histamine 2 receptor antagonists. The use of PPIs is recommended in critically ill patients who have several risk factors for bleeding.^[1,3]^ Moreover, a significant reduction in clinically important gastrointestinal (GI) bleeding in the intensive care unit (ICU) has been reported.^[4]^

The American Society of Health-System Pharmacists (ASHP) recommends against the use of SUP in non-critically ill patients unless the patient has existing risk factors for bleeding.^[1]^ Similarly, the 2024 Society of Critical Care Medicine and ASHP guidelines for prevention of stress-related GI bleeding in critically ill adults recommend stopping SUP once the patient is no longer critically ill or the bleeding risk factor has resolved.^[3]^ However, in real practice, the use of such therapy has also been extended to general medical wards, although the risk of GI bleeding is often low.^[5,6]^ Moreover, healthcare providers continue to employ PPIs as SUP, which is often continued beyond hospital discharge.^[6,7]^ Several studies have suggested that PPIs are being used inappropriately in non-critically ill patients, which may increase the risk of adverse events such as *Clostridium difficile* infection, pneumonia, and fractures, in addition to increased healthcare costs.^[5–7]^

Several surveys have identified the knowledge, beliefs, and attitude of physicians toward SUP in non-critically ill patients.^[5,8,9]^ For instance, a study conducted in USA by Hussain et al found that 9% of physicians prescribed SUP to >25% of their patients, while <50% of the physicians surveyed cited any side effects of PPIs.^[5]^ Another study by Koczka et al found that all medical residents preferred prescribing PPIs, compared to 85% of attending physicians, and 15% of all respondents believed that PPIs were harmless.^[9]^ Moreover, a study by Korayem et al conducted in a single center in Saudi Arabia found that prescriber knowledge scores were low (mean 47%), while no differences by seniority were observed.^[10]^

SUP use is quite prevalent in the Saudi healthcare system in the general wards, including its inappropriate use, as reported by several studies.^[10–13]^ To our knowledge, only a few of these studies were carried out in Saudi Arabia to assess physician knowledge and attitude toward such use.^[10]^ Hence, we aimed to assess the knowledge and attitudes of physicians, including medical residents, toward the use of SUP in non-critically ill patients and to evaluate the factors influencing their prescription practices.

## 2. Methods

### 2.1. Study design, data, and settings

We conducted an electronic cross-sectional survey of physicians and medical residents currently registered with the Saudi Commission for Health Specialties (SCFHS). The survey was distributed among the study cohort from May to July 2021.

### 2.2. Ethical review

This study was managed in accordance with institutional protocols. Ethical approval was obtained from the Regional Research Ethics Committee of the Ministry of Health, Saudi Arabia, and the study was registered with the National Committee of Bio and Medical Ethics (Registration No. H-04-Q-001). Informed consent was not required as the study involved an anonymous physician survey with no patient data collected.

### 2.3. Survey development, distribution, and collection

We developed an online survey by employing a web-based application from SurveyMonkey Inc (San Mateo) as the survey platform and distributed it to eligible participants.^[14]^ The physicians and medical residents currently registered with the SCFHS were identified through the SCFHS database and a total of 3 email reminders were sent to each potential respondent. We utilized an email-based electronic survey distributed via SCFHS as it provided an efficient approach to reach physicians and residents from diverse specialties, experience levels, and geographic regions across Saudi Arabia. This approach ensured broad national coverage and minimized geographic bias. The survey consisted of 15 questions in total and was adopted from previously published studies and modified to suit the targeted cohort (Saudi physicians).^[5,8,9]^ The questionnaire consisted of queries related to demographic information, prescription patterns and frequency, preferred medications, and attitudes toward SUP (Supplementary Document 1, Supplemental Digital Content, https://links.lww.com/MD/Q773).

### 2.4. Measurements and statistical analysis

Descriptive statistics were utilized to present the baseline characteristics of the survey respondents. Continuous variables are presented as means (± standard deviation), while categorical variables are presented as counts and percentages. Exploratory analyses were performed to assess the association of SUP prescription patterns and frequency with years of experience, belief in evidence-based practice, fear of GI bleeding, and the medical rank of the respondents. Chi-squared or Fisher exact tests were used for the analysis where appropriate, with a *P*-value of <.05 considered statistically significant. All analyses were conducted using Stata software version 16.1 (Stata Corp, College Station).

## 3. Results

### 3.1. General characteristics of respondents

A total of 250 physicians participated in this study, of which 210 fully completed the survey. The mean age was 30.8 years (SD = 8.6), and more men (60.9%) responded to the survey (Table 1). In terms of medical rank, the largest group of respondents were residents, followed by consultants, specialists, and fellows. Regarding years of experience, most of the respondents (70%) were in the 1- to 4-years category, followed by the 5- to 10-years categories, and only 12.4% had more than 10 years of experience. The specialty distribution demonstrated a broad representation of medical disciplines. A total of 29% of respondents (n = 61) were internal medicine physicians, 17.6% (n = 37) were family medicine physicians, and 11.4% (n = 24%) were general practitioners (Table 1).

**Table 1:** General characteristics of respondents (N = 210).

Variables	
Age, years (Mean ± S.D)	30.8 (8.6)
Gender distribution, n (%)	Male: 128 (60.9)
Medical rank, n (%)	Consultant: 21 (10)
	Specialists: 20 (9.5)
	Fellows: 7 (3.3)
	Resident: 159 (75.7)
	Others: 3 (1.4)
Years of experience, n (%)	1 to 4 yr: 147 (70)
	5 to 10 yr: 37 (17.6)
	More than 10 yr: 26 (12.4)
Specialties, n (%)	General practice: 24 (11.4)
	Internal Medicine: 61 (29)
	Family Medicine: 37 (17.6)
	Emergency Medicine: 24 (11.4)
	Neurology: 22 (10.5)
	Other: 42 (20)
Do you prescribe stress ulcer prophylaxis for non-critically ill patients (e.g., for patients in general medical wards or internal medicine)?	Yes: 101 (48.1)
	No. 109 (51.9)
How often do you prescribe stress ulcer prophylaxis for non-critically ill patients (e.g., for patients in general medical wards or internal medicine)?	Always: 22 (23.2)
	Often: 28 (29.5)
	Sometimes: 42 (44.2)
	Rarely: 2 (2.1)
	Never: 1 (1.1)
Which one of the following classes would you prefer as stress ulcer prophylaxis?	Proton pump inhibitors: 91 (95.8)
	Histamine 2 receptor antagonists: 3 (3.2)
	Other: 1 (1.1)
Which one of the following medications would you prescribe most often as stress ulcer prophylaxis for non-critically ill patients (non-ICU patients)?	Omeprazole: 58 (61.1)
	Esomeprazole: 27 (28.4)
	Pantoprazole: 9 (9.5)
	Ranitidine: 1 (1.1)
Which one of the following routes of administration do you prefer when prescribing omeprazole as stress ulcer prophylaxis for non-critically ill patients? (For patients who have no contraindication to take oral medications)	Oral omeprazole: 81 (85.3)
	Intravenous omeprazole: 14 (14.7)

ICU = intensive care unit, N = numbers, n = numbers, SD = standard deviation.

### 3.2. SUP prescription practices

When asked whether they prescribed SUP for non-critically ill patients, the responses of the study cohort were divided. A total of 48.1% (n = 101) reported that they prescribed SUP, while 51.9% (n = 109) did not. Regarding prescription frequency, 23.2% (n = 22) stated that they prescribed SUP always, while 29.5% (n = 28) prescribed it often, followed by 44.2% (n = 42) who prescribed it sometimes. Fewer respondents reported prescribing SUP rarely (2.1%, n = 2) or never (1.1%, n = 1) (Table 1). Physicians preferred prescribing PPIs over other medications (95.8%, n = 91). Among specific agents, omeprazole (61.1%, n = 58) was the most prescribed, followed by esomeprazole (28.4%, n = 27). When asked about the preferred route of administration for omeprazole, the respondents stated that the oral route was preferred (85.3%, n = 81).

### 3.3. Evidence-based use of SUP

**Figure 1.**
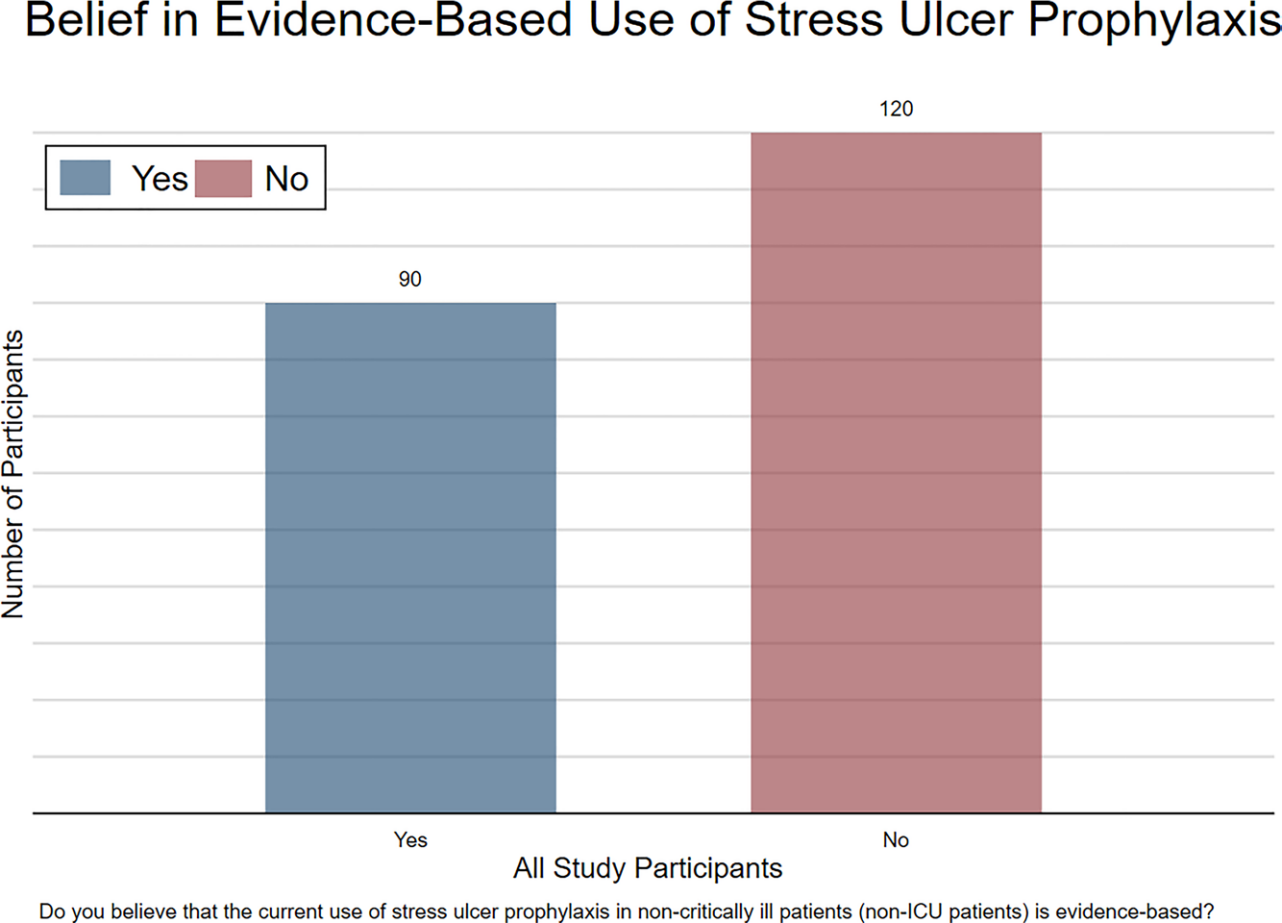
Belief in evidence-based use of SUP among all study participants. Bar chart showing the number of participants who believe that the current use of stress ulcer prophylaxis in non-critically ill patients (non-ICU patients) is evidence-based. ICU = intensive care unit, SUP = stress ulcer prophylaxis.

**Figure 2.**
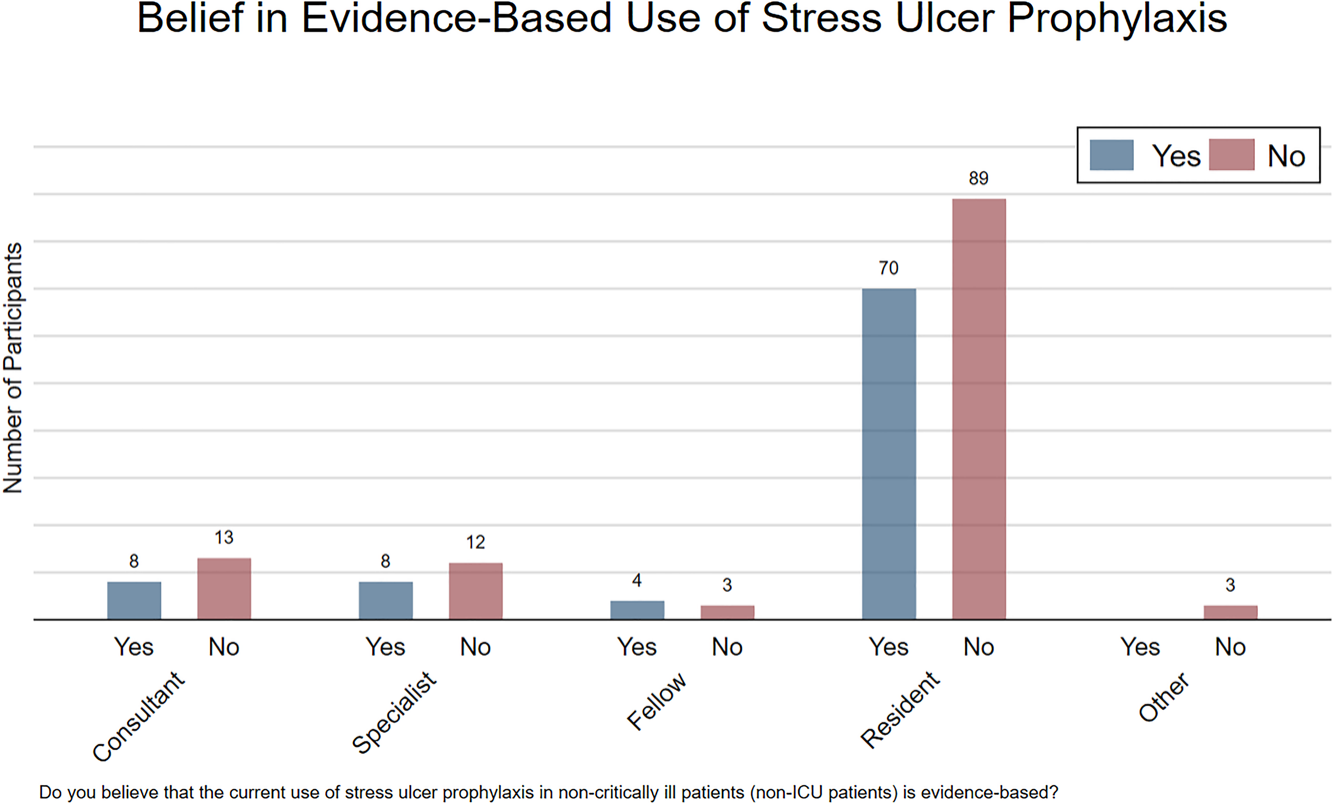
Belief in evidence-based use of SUP by medical rank. Stratified bar chart showing responses to whether current use of stress ulcer prophylaxis in non-ICU patients is evidence-based, categorized by professional role. ICU = intensive care unit, SUP = stress ulcer prophylaxis.

Among the respondents, 42.8% (n = 90) believed that the current use of SUP in noncritically ill patients was evidence-based, whereas 57.2% (n = 120) disagreed with this statement (Fig. 1). When explored by medical rank, 8 consultants, 8 specialists, and 4 fellows answered in the affirmative. Among the residents, who comprised most of the respondents, 70 out of 159 answered in the affirmative (Fig. 2).

### 3.4. Main reasons for prescribing SUP

**Figure 3.**
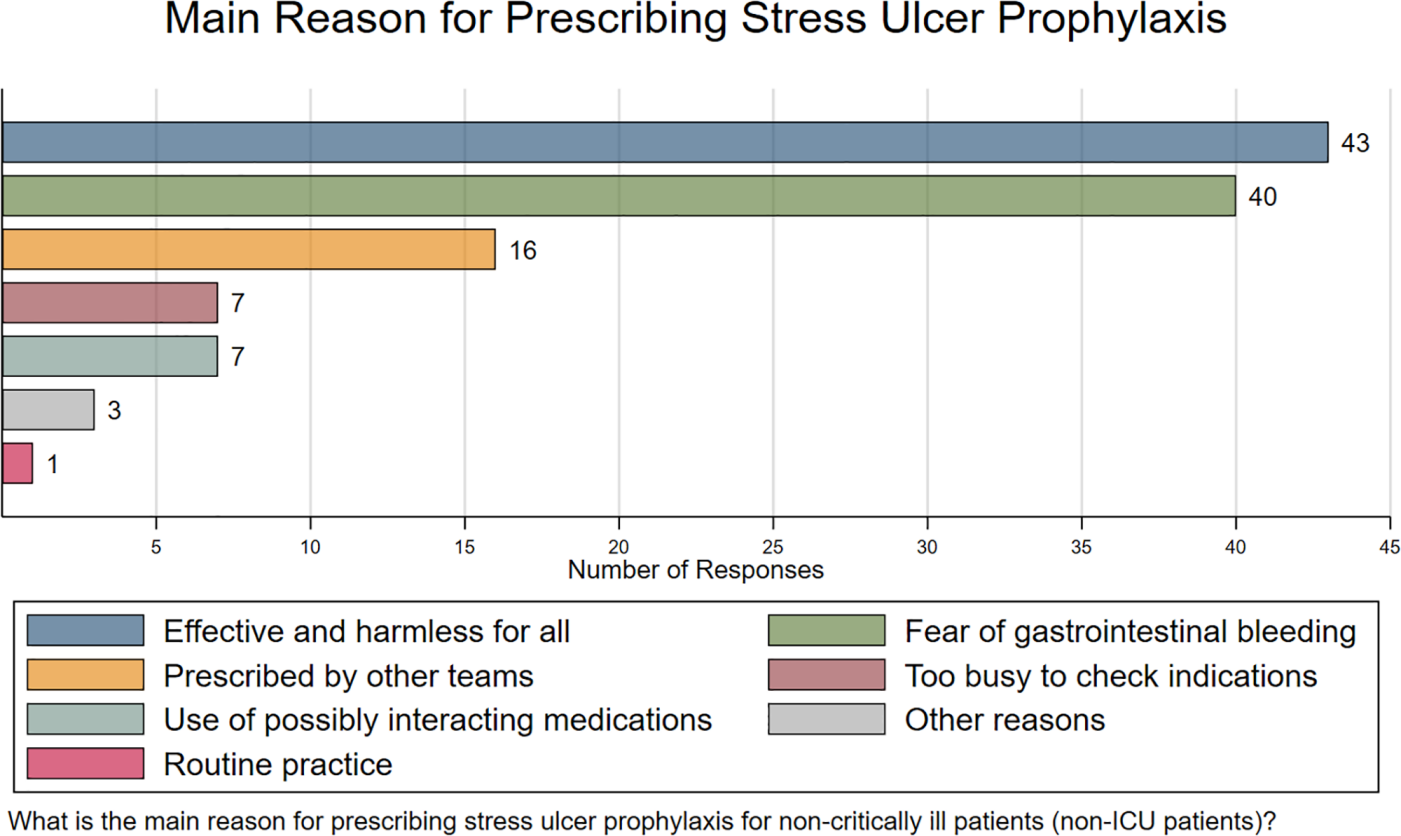
Main reason for prescribing stress ulcer prophylaxis in non-ICU patients. Horizontal bar chart summarizing participants’ responses to the question on the main reason for prescribing SUP. ICU = intensive care unit, SUP = stress ulcer prophylaxis.

The reasons for prescribing SUP are shown in Figure 3. The most cited reasons were “SUP is effective and harmless for all,” followed by “fear of GI bleeding.” The former was cited 43 times, whereas the latter was cited 40 times, followed by “prescribed by other teams,” “too busy to check indications,” and “use of possibly interacting medications,” which they were cited 16, 7, and 7 times, respectively.

### 3.5. Pharmacist recommendations and discontinuation of SUP

**Figure 4.**
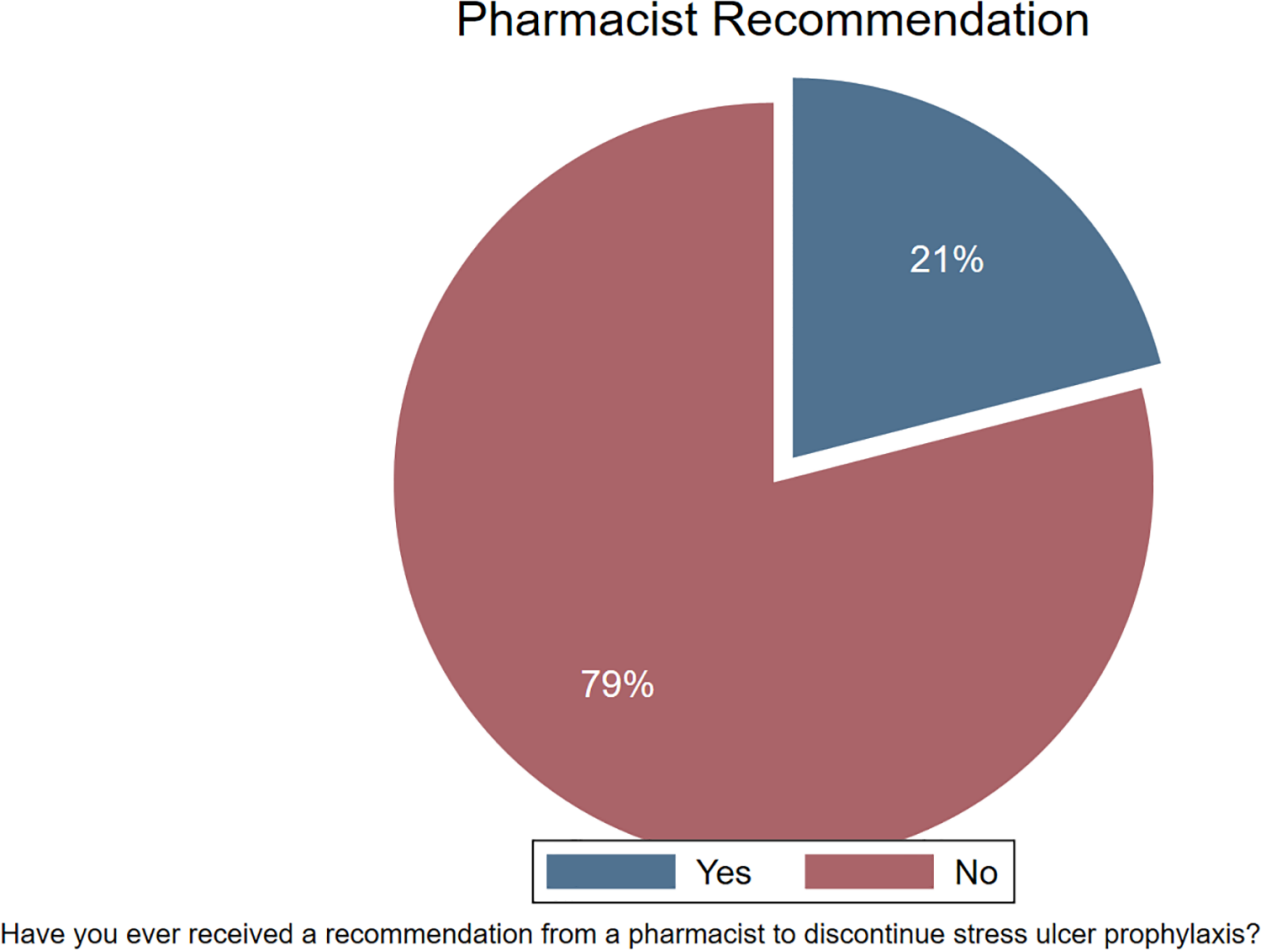
Receipt of pharmacist recommendation to discontinue stress ulcer prophylaxis. pie chart illustrating the percentage of participants who have received a recommendation from a pharmacist to discontinue SUP. SUP = stress ulcer prophylaxis.

**Figure 5.**
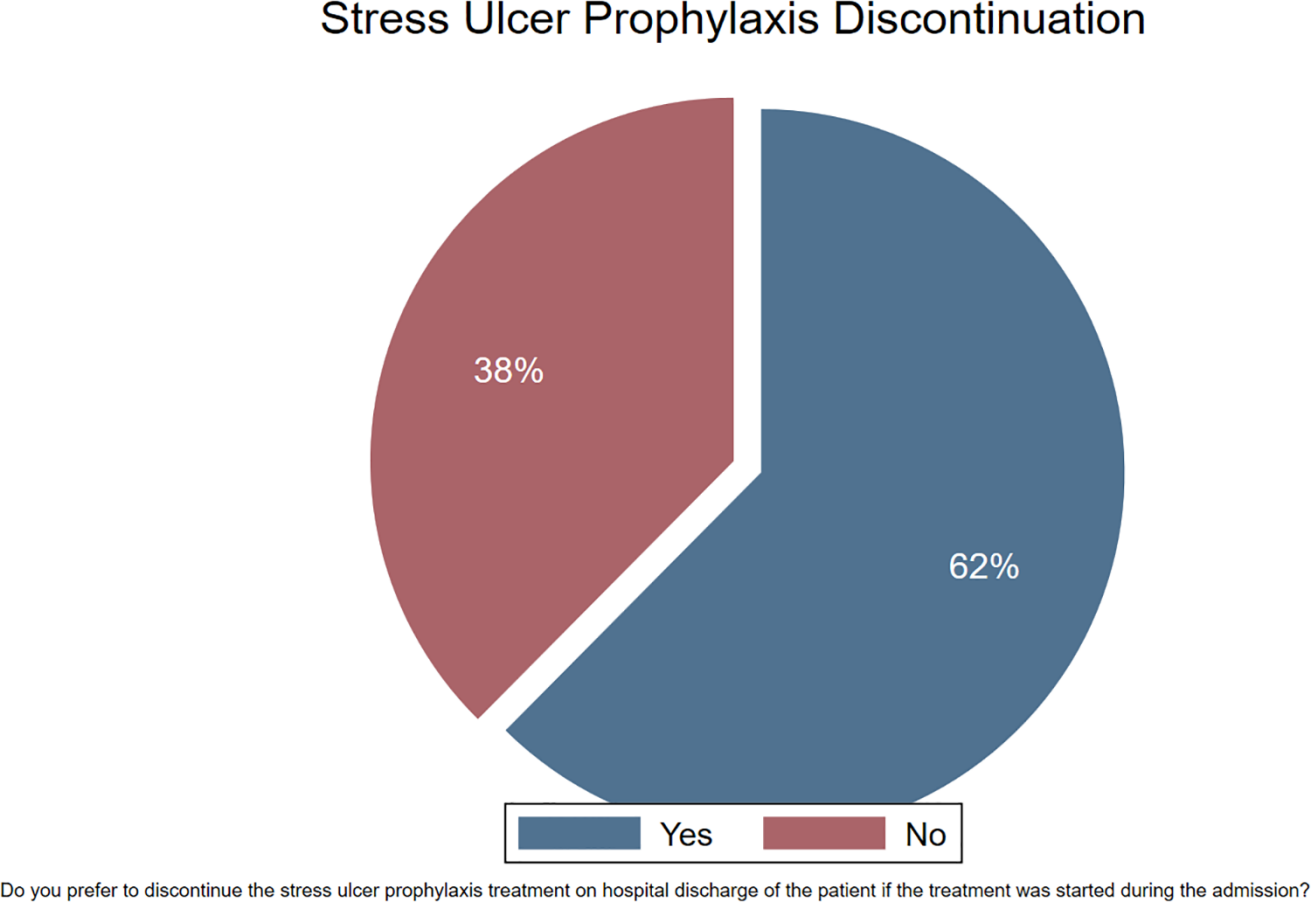
Discontinuation of SUP on hospital discharge. Pie chart showing preferences for discontinuing stress ulcer prophylaxis at hospital discharge. SUP = stress ulcer prophylaxis.

Most respondents (79%) did not receive a pharmacist recommendation to discontinue SUP (Fig. 4). Similarly, 62% of the respondents stated that they would discontinue SUP use upon hospital discharge (Fig. 5).

### 3.6. Factors associated with SUP prescription

Table 2 summarizes the factors strongly associated with SUP prescription. There was a statistically significant association between SUP prescription and years of clinical experience (χ² = 7.89, *P* = .019), belief in evidence-based use (χ² = 24.44, *P* <.001), fear of GI bleeding (χ² = 53.33, *P* <.001), and medical rank (χ² = 11.25, *P* = .024).

**Table 2:** Exploratory associations between SUP prescription and years of experience, belief in evidence-based use, fear of GI bleeding, and medical specialty.

Association	Chi-square value	P-value
SUP prescription and years of experience	7.89	.019
SUP prescription and belief in evidence-based use	24.44	.000
SUP prescription and fear of GI bleeding	53.33	.000
SUP prescription and medical rank	11.25	.024

GI = gastrointestinal, SUP = stress ulcer prophylaxis.

## 4. Discussion

This cross-sectional survey aimed to assess the knowledge and attitudes of physicians toward SUP use for non-critically ill hospitalized patients. Our key findings indicated that approximately half of the physicians prescribed SUP, while more than half reported that they prescribed it either always or often. A significant proportion of respondents (42.8%) believed that the current use of SUP in non-critically ill patients was evidence-based. Additionally, the most frequently cited reasons for SUP use were the belief of respondents that SUP is effective and harmless for everyone and their fear of GI bleeding. Most respondents did not receive recommendations from pharmacists regarding SUP discontinuation, and 38% continued it upon hospital discharge. Further, SUP use was significantly associated with years of clinical experience, belief in evidence-based use, fear of GI bleeding, and medical rank.

Previous observational studies have shown that SUP use, including its inappropriate use, is prevalent in non-critically ill patients.^[15–19]^ In Saudi Arabia, the inappropriate use of SUP in the medical and surgical wards has ranged from 30.2% to 96.4%.^[10,11,13]^ In the present study, when respondents were asked if they prescribed SUP, 48.1% (n = 101) answered in the affirmative, while more than half stated that they would prescribe it always or often. Compared with other survey-based studies, Koczka et al reported that 48% of residents and 56% of attendants agreed that most patients were started on SUP upon admission to a medical ward.^[10]^ Similarly, Hussain et al reported that 69% of physicians prescribed SUP to more than 25% of their non-ICU patients, and 18% prescribed SUP to more than 75% of their patients.^[5]^ Korayem et al found that 76.3% of physicians reported prescribing SUP.^[10]^

In the current study, 42.8% of respondents believed that the current use of SUP in non-critically ill patients was evidence-based. Similar to the results of the present study, Hussain et al found that 41% (n = 40) of physicians believed that SUP use is evidence-based.^[5]^ Moreover, only 23% reported that they would prescribe SUP based on clinical guidelines.^[5]^ Koczka et al found that 41% of attending physicians and 30% of residents believed that SUP was indicated.^[9]^ Our present findings are consistent with previous reports, which indicated that a significant proportion of physicians believe in SUP use in non-ICU settings, and that this is in line with its overuse in real practice.

In our study, PPIs were predominantly (95.8%) preferred by physicians as SUPs, consistent with previously published data.^[5,9,10]^ Numerous factors may play a role in the choice of PPIs including medication availability, settings (surgical vs medical ward), and physician preferences. For instance, most Ministry of Health hospitals in Saudi Arabia use omeprazole, which partly explains why 61.1% of the survey responders used omeprazole, followed by esomeprazole at 28.4%. In terms of differences among PPIs, no data are available for 1 medication being superior to another in general medical wards. For instance, Liu et al conducted a systematic review and network meta-analysis of non-ICU patients and found no significant differences between specific PPI agents (omeprazole, pantoprazole, lansoprazole, and esomeprazole).^[20]^

Studies have listed various reasons for SUP prescriptions. Hussain et al reported fear of GI bleeding and litigation as the main barriers,^[5]^ while Koczka et al reported habitual practices and misbeliefs about safety.^[9]^ Conversely, Korayem et al cited the absence of protocols, prescriber workload, and the perception that SUP is harmless as the main factors.^[10]^ In our study, the most cited reasons for prescribing SUP were the belief that it is effective and harmless for all, followed by a fear of GI bleeding, which is in line with previous reports. Moreover, years of clinical experience, belief in evidence-based SUP use, fear of GI bleeding, and medical rank were significantly associated with SUP use.

The unnecessary continuation of SUP after hospital discharge remains a significant issue. It has been previously reported that the prevalence of inappropriate continuation has ranged from 24 to 44.7%.^[21–24]^ Moreover, in 1 study, out of those inappropriately discharged on SUP, 64% continued to receive the PPI at their first outpatient visit.^[21]^ Further, some factors were found to be associated with extended use, such as upper endoscopy, admission to the surgical ward, long hospital stay, and discharge to a nursing home.^[21,22]^ In our study, 38% of respondents stated that they would continue SUP upon hospital discharge, which is considered a significant proportion indicating that this issue exists in Saudi hospitals.

Notably, 75.7% of the respondents in our study were medical residents, whereas this percentage was 67% in Hussain et al,^[5]^ 61% in Koczka et al,^[9]^ and Korayem et al^[10]^ did not specify this number. This is important to consider when interpreting survey results, especially because medical residents are most likely to fill out those surveys, possibly due to time constraints for those with higher medical ranks. Moreover, it is possible that residents may often overestimate the risk of GI bleeding in non-ICU patients, and their lack of knowledge and prescription habits may have influenced our current findings. These results emphasize the need for practice-based educational interventions directed at different healthcare providers.

Liberman et al conducted a prospective pre- and post-intervention cohort study to investigate the impact of a practice-based educational intervention on reducing inappropriate SUP prescriptions among internal medicine residents.^[25]^ The 1-hour educational session included evidence relating to prescribing SUP based on ASHP guidelines, prescription data and feedback from residents themselves, healthcare cost analysis, discussion of medication side effects, and appropriate indications. This study found that such targeted educational sessions led to a significant reduction in the inappropriate use of SUP, with effects that are sustainable and transferable to new trainees.^[25]^ Moreover, in addition to education, it is important to combine such efforts with stewardship programs and decision-support tools that ensure evidence-based prescriptions and efforts toward improving institutional culture.^[5,9,10]^ Further, it is important to address the fear of GI bleeding by presenting data on its low incidence and assessing patient risk by employing the appropriate tools and clinical guidelines. Malhis et al conducted a prospective observational study in Saudi Arabia using ASHP guidelines for SUP and the Herzig clinical risk score for nosocomial GI bleeding risk as assessment tools.^[13]^ The study found that 96.4% of those who received SUP in general wards had low-risk bleeding as assessed by the Herzig score.^[13]^

During transition of care (ICU to floor or floor to discharge), inappropriate SUP continuation occurs, and efforts such as medication reconciliation and targeted interventions may help reduce unnecessary SUP use. Studies have suggested that pharmacists should be actively involved in medication reconciliation during care transition.^[26,27]^ A systematic review of 8 studies found that pharmacist-led interventions have significantly reduced inappropriate use of SUP, including at transfer from ICU to medical wards or at hospital discharge.^[27]^ Utilized interventions included employing clinical guidelines, educational campaigns, reviewing medications during medical rounds, and providing recommendations for SUP changes. This led to a reduction in medication costs.^[27]^ In our study, most respondents did not receive recommendations from pharmacists regarding the discontinuation of SUP. We did not investigate whether this was due to a lack of clinical pharmacists at the institution or their limited involvement. Nonetheless, pharmacist and interdisciplinary team involvement are required.

The Saudi Ministry of Health published a national protocol in November 2021 regarding SUP for both ICU and non-ICU adult patients.^[28]^ The purpose of this protocol was to guide healthcare practitioners in the appropriate use of SUP by providing a structured evidence-based approach. This protocol also aimed to empower pharmacists to discontinue inappropriate SUP, communicate with prescribers, promote stewardship, and adhere to clinical guidelines. While this step was a positive development, adherence to it will hopefully increase over time with greater awareness and implementation of the guidelines. More well-designed national studies of adequate size and power are needed to assess the impact of the protocol and identify opportunities for improvement.

This study has some limitations. Our study did not collect information on potential confounders, including institutional protocols, guideline variations, patient population characteristics, and physicians’ previous training or clinical exposure, all of which may influence SUP prescribing patterns. As a result, observed differences in reported practices may partly reflect these unmeasured factors rather than individual decision-making alone. In addition, responses were self-reported and may be subject to recall bias, which could affect the accuracy of reported practices. In this study, we did not assess the knowledge scores of the physicians or their beliefs about the cost of SUP. Although the survey was distributed to all physicians registered at the SCFHS, we could not provide the response rate; additionally, since most respondents were male medical residents, these results cannot be generalized to all physicians who did not participate in the study.

## 5. Conclusions

This cross-sectional survey revealed important insights into the knowledge and attitudes of physicians toward prescribing SUP for non-critically ill patients in Saudi Arabia. A significant proportion of the responding physicians believed that SUP use was an evidence-based practice and reported prescribing it regularly or frequently. Important reasons cited by physicians included belief in PPI safety for all patients and fear of GI bleeding. Our present findings point to opportunities for improved education and interprofessional support (especially with participating pharmacists) to enhance SUP prescriptions, including reducing its inappropriate use and unnecessary continuation upon discharge.

## Acknowledgments

The Researchers would like to thank the Deanship of Graduate Studies and Scientific Research at Qassim University for financial support (QU-APC-2025).

## Author contributions

**Conceptualization:** Osamah M. Alfayez, Abdullah A. Alalwan.

**Data curation:** Mashael AlFaifi, Yara Ajeebi.

**Formal analysis:** Omar S. Alkhezi.

**Methodology:** Abdullah A. Alalwan, Omar S. Alkhezi.

**Supervision:** Osamah M. Alfayez.

**Writing – original draft:** Osamah M. Alfayez, Eiman Ibrahim.

**Writing – review & editing:** Osamah M. Alfayez, Mashael AlFaifi, Abdullah A. Alalwan, Eiman Ibrahim, Omar S. Alkhezi.

## Supplementary Material


