# Supplementary material for: Stress ulcer prophylaxis in non-critically ill patients: A cross-sectional survey in Saudi Arabia
Source: Medicine (Baltimore). 2025 Nov 28;104(48):e46252. doi: 10.1097/MD.0000000000046252 (PMC12662385; doi:10.1097/MD.0000000000046252)
Supplement: Supplementary file 1 [file medi-104-e46252-s001.docx]

1. What is your age? ________
2. What is your gender?
3. Female
4. Male
5. Are you Saudi?
6. Yes
7. No
8. Which one of the following best describes you?
9. Resident
10. Fellow
11. Specialist
12. Consultant/attending
13. Other (Specify: _____________)
14. What is your specialty? _________
15. Which one of the following best describes your years of experience as a physician?
16. 1-4 years
17. 5 to 10 years
18. >10 years
19. Do you prescribe stress ulcer prophylaxis to non-critically ill patients (e.g., for patients in general medical wards or internal medicine)?
20. Yes
21. No
22. How often do you prescribe stress ulcer prophylaxis to non-critically ill patients (e.g., for patients in general medical wards or internal medicine)?
23. Always
24. Often
25. Sometime
26. Rarely
27. Never
28. Do you believe that the current use of stress ulcer prophylaxis in non-critically ill patients (non-ICU patients) is evidence-based?
29. Yes
30. No
31. Which one of the following classes would you prefer as stress ulcer prophylaxis?
32. Proton Pump Inhibitors (PPIs) such as omeprazole or esomeprazole
33. Histamine 2 Receptor Antagonists (H_2_ Blockers) such as cimetidine or famotidine
34. Other (Specify: __________)
35. Which one of the following medications would you prescribe most often as stress ulcer prophylaxis to non-critically ill patients (non-ICU patients)?
36. Esomeprazole (Nexium)
37. Omeprazole (Prilosec)
38. Pantoprazole (Pantomax)
39. Lansoprazole (Prevacid)
40. Cimetidine (Tagamet)
41. Famotidine (Zantac)
42. Which one of the following routes of administration do you prefer when prescribing omeprazole as stress ulcer prophylaxis for non-critically ill patients?
43. Oral omeprazole
44. Intravenous (IV) omeprazole
45. N/A
46. What is the main reason for prescribing stress ulcer prophylaxis to non-critically ill patients (non-ICU patients)?
47. I fear that the patient might develop gastrointestinal bleeding.
48. I think stress ulcer prophylaxis therapy is effective and harmless to all patients
49. I am too busy to check for the indication of stress ulcer prophylaxis therapy
50. Usually, it’s prescribed by another medical team or in the emergency department, and I am reluctant to discontinue the medication
51. Other (Specify: ____________________)
52. Have you ever received a recommendation from a pharmacist to discontinue stress ulcer prophylaxis (e.g., omeprazole)?
53. Yes
54. No
55. Do you prefer to discontinue the stress ulcer prophylaxis treatment on hospital discharge of the patient if the treatment was started during the admission?
56. Yes
57. No
